# Composition and distribution of lice (Insecta: Phthiraptera) on Colombian and Peruvian birds: New data on louse-host association in the Neotropics

**DOI:** 10.3897/BDJ.6.e21635

**Published:** 2018-08-28

**Authors:** Juliana Soto-Patiño, Gustavo A Londoño, Kevin P Johnson, Jason D Weckstein, Jorge Enrique Avendaño, Therese A Catanach, Andrew D Sweet, Andrew T Cook, Jill E Jankowski, Julie Allen

**Affiliations:** 1 Universidad Pedagógica y Tecnológica de Colombia, Tunja, Colombia Universidad Pedagógica y Tecnológica de Colombia Tunja Colombia; 2 Departamento de Ciencias Biológicas, Facultad de Ciencias Naturales, Universidad Icesi, Cali, Colombia Departamento de Ciencias Biológicas, Facultad de Ciencias Naturales, Universidad Icesi Cali Colombia; 3 Illinois Natural History Survey, Prairie Research Institute, University of Illinois, Urbana-Champaign, Champaign, IL, United States of America Illinois Natural History Survey, Prairie Research Institute, University of Illinois Urbana-Champaign, Champaign, IL United States of America; 4 Department of Ornithology, Academy of Natural Sciences and Department of Biodiversity, Earth, and Environmental Science, Drexel University, Philadelphia, United States of America Department of Ornithology, Academy of Natural Sciences and Department of Biodiversity, Earth, and Environmental Science, Drexel University Philadelphia United States of America; 5 Laboratorio de Biología Evolutiva de Vertebrados, Departamento de Ciencias Biológicas, Universidad de los Andes, Bogotá, Colombia Laboratorio de Biología Evolutiva de Vertebrados, Departamento de Ciencias Biológicas, Universidad de los Andes Bogotá Colombia; 6 Department of Biological Sciences, University of Alberta, Alberta, Canada Department of Biological Sciences, University of Alberta Alberta Canada; 7 Biodiversity Research Centre, Department of Zoology, University of British Columbia, Vancouver, BC, Canada Biodiversity Research Centre, Department of Zoology, University of British Columbia Vancouver, BC Canada; 8 Department of Biology, University of Nevada, Reno, United States of America Department of Biology, University of Nevada Reno United States of America

**Keywords:** Ectoparasites, Feather Lice, Tropical Forests

## Abstract

The diversity of permanent ectoparasites is likely underestimated due to the difficulty of collecting samples. Lice (Insecta: Phthiraptera) are permanent ectoparasites of birds and mammals; there are approximately 5,000 species described and many more undescribed, particularly in the Neotropics. We document the louse genera collected from birds sampled in Peru (2006–2007) and Colombia (2009–2016), from 22 localities across a variety of ecosystems, ranging from lowland tropical forest and Llanos to high elevation cloud forest. We identified 35 louse genera from a total of 210 bird species belonging to 37 avian families and 13 orders. These genera belong to two suborders and three families of lice: Amblycera, families Menoponidae (present on 131 bird species) and Ricinidae (39 bird species); and Ischnocera, family Philopteridae (119 bird species). We compared our bird-louse associations with data in Price et al. (2003) and recently published Neotropical studies. The majority of bird-louse associations (51.9%) were new, with most of these coming from Passeriformes, the most diverse avian order, with the most poorly known louse fauna. Finally, we found geographical variation in louse infestation and prevalence rates. With this study, we report the first comprehensive documentation of bird-louse associations for Colombia and substantially increase the known associations documented for Peru.

## Introduction

Parasites are one of the most common forms of life on the planet (Price 1980). They have evolved repeatedly in every major clade (Poulin and Morand 2000). Although parasites are amongst the most diverse organisms in the world, few are well studied. Permanent ectoparasites are particularly difficult to study because they live their entire life cycle on hosts (Marshall 1981) and require capturing the host to sample them.

Lice (Insecta: Phthiraptera) are permanent parasites occurring on both birds and mammals. There are approximately 5,000 described species of lice, about 3,000 of which are known from birds (Price et al. 2003, Smith et al. 2011). The taxonomic diversity of lice is positively correlated with the taxonomic diversity of their hosts (Eichler 1942, Vas et al. 2012). Colombia and Peru harbor the richest avifaunas in the world (Jetz et al. 2012), with 1,878 and 1,852 bird species, respectively (Avendaño et al. 2017), and, correspondingly, the highest diversity of avian lice is thought to be found in these regions (e.g. Valim and Weckstein (2013)). Currently, however, there is limited knowledge of louse-host associations and louse diversity from these countries (e.g. Clayton et al. (1992) and the Neotropics in general (Clayton et al. (1992), Marini et al. (1996), Valim and Weckstein (2013). This is due in part to the poor representation of louse specimens in museum collections and the lack of louse specialists and field workers who sample parasites when collecting or handling birds. Therefore, the diversity of known louse species at regional scales is not on par with lists of avian host diversity from these countries. Our main objective is to provide novel information about the composition and distribution of lice on Colombian and Peruvian birds.

From large collections of louse specimens from birds in Peru, Clayton et al. (1992) and Clayton and Walther (2001) examined how host ecology and morphology influence louse diversity across a sample of 127 bird species. These two studies, amongst other taxonomic studies published using the same specimens e.g. Price and Clayton (1995), Price and Clayton (1989), provide most of the known louse-host associations from Peruvian birds. Much less information is available for Colombia, apart from the work of Melbourne A. Carriker (1879–1965), who collected mostly non-passerine birds and their associated lice and a study by Parra-Henao et al. (2011) where they identified lice from 18 bird species from the Cordillera Central near Medellín (Valle de Aburrá). Although this previous work provides an excellent starting point for understanding the diversity of lice in the Neotropics, the numbers of birds examined for lice is a small sample of the total avian diversity in this region.

In this study, we provide data from extensive sampling and description of louse-host associations from Colombia and Peru. Material was collected from 22 localities over nine years. From these samples, we identified 36 unique genera of lice and compared our results with those found in previous studies and with data compiled in the published checklist in Price et al. (2003). We found that over 50% of the louse-host associations were previously unreported and suggest that further data from these collections will be important to identify factors associated with louse diversity in the Neotropics. The data presented here provide the foundation for a long-term project sampling louse diversity across the Andes. This dataset will provide the basis for answering large-scale questions about patterns of diversity along elevational, habitat and host taxonomic gradients. The long-term project will include species level identification, taxonomic description and exploration of macro-ecological patterns along with archiving and storage of louse specimens.

## Material and methods

Lice were collected at 22 localities in Peru (2006–2007) and Colombia (2009–2016) (Table 1). In Peru, samples were collected by GAL and JEJ at four stations from Andean foothill forest (800 m a.s.l.) to high elevation cloud forest (3,000 m a.s.l.) inside Manu National Park or its buffer zone along a contiguously forested altitudinal gradient (Fig. 1a). In Colombia, samples were collected by GAL, JEA and JSP at 18 sites across the country, which ranged in elevation and habitat from 100 m a.s.l. to 2,800 m a.s.l., including savannah and gallery forest, lowland tropical forest and humid premontane and montane cloud forest (Fig. 1b).

At each site, 10 to 20 netting stations were run and, at each station, 10 mist nets were opened for three days to capture birds. Each netting station was sampled twice during each 4 to 6 month field season. After removing birds from the nets, each individual host was placed in a clean cloth bag until processing for ectoparasites. We used three methods for collecting ectoparasites, detailed in Clayton and Drown (2001): 1) Post-mortem ruffling, 2) visual examiniation and, for the majority of samples 3) dust-ruffling. To dust-ruffle the birds, we applied ~1 ml of EverGreen pyrethrum dust (McLaughlin Gormley King Company, MN, USA) to captured birds and then ruffled feathers from all body regions except the head. Five minutes after we applied the powder, we ruffled each bird's feather tracts over a plastic sheet for 30 to 60 seconds to remove powder and ectoparasites. We transferred all powder and ectoparasites that fell on to the sheet to a 1.5ml Eppendorf tube filled with 96% ethyl alcohol and inserted a label with host metadata.

JEA also collected ectoparasite specimens using Clayton and Drown's (2001) post-mortem ruffling method for euthanised avian hosts. These hosts were collected and prepared as museum voucher specimens. To collect ectoparasites, JEA placed each euthanised host in a Ziploc bag with cotton soaked in ethyl acetate for 20 minutes. He then removed the bird from the bag and ruffled the plumage for 60 seconds over a white sheet of paper. Each specimen was returned to its Ziploc bag (with cotton soaked with ethyl acetate), ruffling the plumage two additional times, at intervals of 15 minutes. The ectoparasites were collected from the paper with a small brush and placed in a vial with 96% ethyl alcohol with a label including host specimen metadata. Bird voucher specimens were deposited in the bird collection of Instituto de Ciencias Naturales (ICN) of Universidad Nacional (Bogotá, Colombia) and the Museum of Natural History (MHNU) at Universidad de los Llanos (Villavicencio, Meta, Colombia). Lice were separated from the other ectoparasites, placed into individual vials and identified to genus using taxonomic keys Price et al. (2003). Host taxonomy followed the South American Classification Committee Remsen et al. (2017). Many louse species require microscopic examination of a slide-mounted specimen for species level identification. As this will be the focus of future work, these lice were only identified to genus. All specimens are stored at -80C for later DNA extraction and slide mounting at the Universidad Icesi, Cali Colombia. Vouchered, slide-mounted specimens will be made available at Universidad Icesi in Colombia and The Museum of Natural History at the University of Nevada, Reno in the U.S. The Colombian permit was approved by the ANLA by the Resolución 509 del 21 de mayo del 2014 and the Peruvian permit was approved by the Institutional Animal Care and Use Committee at the University of Florida (Protocol #: 201106068) and by permits from the government of Peru (0239-2013 MINAGRI-DGFFS/DGEFFS 2013).

We compared our findings with the world checklist of chewing lice in Price et al. (2003) and recently published taxonomic literature on Neotropical lice in Price et al. (2005), Price et al. (2008), Price and Dalgleish (2006), Sychra et al. (2007), Kounek et al. (2011a), Kounek et al. (2011b), Valim et al. (2011). Using these resources, for each host species in our study, we classified the louse fauna documented amongst our samples combined from both Colombia and Peru into one of four categories.

0) Not previously reported - avian species with no louse association data reported.

1) Same as reported - avian species for which our study found the same louse genera as reported.

2) Fewer than reported - avian species for which our study found fewer louse genera than reported

3) More than reported - avian species for which our study found more louse genera than reported

## Results

In Colombia, we sampled 1,032 individual birds from 280 species. Just over half, 51.6% (532), of these birds were infected with ectoparasites (i.e. feather mites, ticks, parasitic flies, fleas and lice) and we found lice on 30% (310) of individual hosts from 138 avian species, 36 avian families and 13 avian orders (Table 2). In Peru, we found lice on 262 individual birds from 98 species, 19 families and 5 orders (Table 3). In both countries combined, we identified 35 louse genera on 210 bird species from 37 avian families and 13 avian orders. Lice documented in this study are from two suborders and three families: Suborder Amblycera (Menoponidae and Ricinidae); and suborder Ischnocera (Philopteridae).

***Suborder Amblycera***

**Menoponidae** - Six menoponid louse genera were distributed on 131 bird species: *Myrsidea* Waterston 1915 (120 bird species), *Menacanthus* Neumann 1912 (8), *PsittacobrosusCarriker 1954* (3), *Machaerilaemus* Harrison 1915 (2), *EureumNitzsch 1818* (1) and *Osborniella* Thompson 1948 (1).

**Ricinidae** – Three ricinid louse genera were distributed on 39 bird species: *Ricinus* De Geer 1778 (36 bird species), *Trochiliphagus* Carriker 1960 (2) and *Trochiloecetes* Paine and Mann 1913 (1).

***Suborder Ischnocera***

**Philopteridae** – Twenty six philopterid genera were distributed on 119 bird species: *PhilopterusNitzsch 1818* (42 bird species), *BrueeliaKéler 1936* (30), *Rallicola* Johnston and Harrison 1911 (18), *Penenirmus* Clay and Meinertzhagen 1938a (7), *Formicaphagus* Carriker 1957 (5), *Picicola* Clay and Meinertzhagen 1938a (4), *Alcedoffula* Clay and Meinertzhagen 1939 (3), *Columbicola* Ewing 1929 (3), *Furnariphilus* Price and Clayton 1995 (2), *MulcticolaClay and Meinertzhagen 1938b* (2), *Physconelloides* Ewing 1927 (2), *AustrophilopterusEwing 1929* (1), *Campanulotes* Kéler 1939 (1), *Cotingacola* Carriker 1956 (1) *CuculoecusEwing 1936* (1), *DegeeriellaNeumann 1906* (1), *Goniodes* Nitzsch 1818 (1), *LipeurusNitzsch 1818* (1) *Oxylipeurus* Mjöberg 1910 (1), *Paragoniocotes* Cummings 1916 (1), *PseudocophorusCarriker 1940* (1), *Rhynonirmus* Thompson 1935 (1), *Strongylocotes* Tachenberg 1882 (1), *Saemundssonia* Timmermann 1936 (1), *SturnidoecusEichler 1944* (1) and *Vernoniella* Guimarães 1942 (1).

In total, including the two louse suborders, 131 bird species had one louse genus, 61 had two louse genera, 16 had three louse genera, 1 had four and 1 had five louse genera.

**Figure 2.**
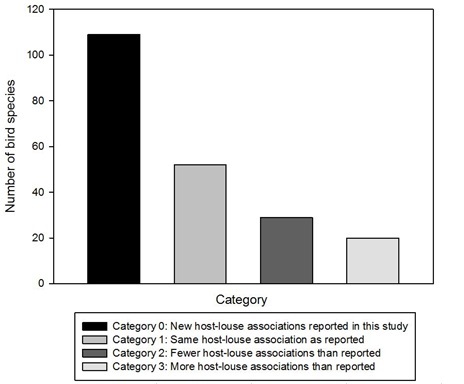
Bird-louse associations included in each category described in the methods above. The Y axis represents the number of bird species and the X axis indicates the categories in which bird species were grouped according to reported louse-bird associations.

We compared our findings with the world checklist of chewing lice in Price et al. (2003) and more recent publications. We report new louse generic associations for 109 of 210 bird species (51.6% of the host species sampled; Tables 2 and 3). For 52 bird species (24.8% of the host species sampled), we found the same number of louse genera as previously reported and, in 29 bird species (13.8% of the host species sampled), we found fewer genera than previously reported. In addition, for 20 bird species (9.5% of the host species sampled), we found more louse genera than previously reported Fig. 2.

## Data resources

The dataset is the result of several trips to 22 localities to study Neotropical bird communities in Colombia and Peru Table 1. In this study, we report lice on a total of 572 individual hosts totalling 210 bird species from 37 avian families and 13 orders. We identified 35 louse genera from two suborders and three families: Suborder Amblycera (Menoponidae and Ricinidae); and suborder Ischnocera (Philopteridae) Suppl. material 1

## Discussion

In the present study, we report the genera of lice collected from 210 bird species at 22 sites in Colombia and Peru. We compared the louse-host association found in our study with the known genera of lice from these species of birds. We used Price et al. (2003), the most complete published bird-louse association list, along with recent Neotropical host-louse faunistic and taxonomic publications to assess the novelty of the host-parasite associations documented by our study.

We report 109 novel host-louse generic associations. This was not unexpected as we sampled several lowland and Andean habitats which have previously had few studies of bird-louse associations.

The majority (87.1%) of these new records were from Passeriformes. Knowledge of lice from many Passeriformes is relatively poor compared to non-passerines Sychra et al. (2007) and thus the diversity and number of undescribed parasites from these hosts is likely high e.g. Valim and Weckstein (2013), as confirmed by recent taxonomic descriptions and new associations of lice from Neotropical birds in the families Tyrannidae Price et al. 2005, Thraupidae [Bibr B3798455][Bibr B3798489], Price and Johnson 2009, Furnariidae Sychra et al. 2007, Parulidae Kounek et al. 2011a and Cardinalidae, Emberizidae and Fringillidae Kounek et al. 2011b. These studies are likely only the beginning of describing new species in these mega-diverse louse groups found on Neotropical passerine birds. For example, Valim and Weckstein (2013) point out that louse genera such as *Myrsidea* harbour large numbers of undescribed species. Our data show that the majority of Passeriformes sampled (64.5%) have *Myrsidea* and many of them are likely to be new species.

The distribution of lice is related to the distribution of their hosts Rózsa and Vas (2015) and many orders and families typically have parasites of distinctive louse faunas Smith (2001). Our data are consistent with generalised patterns across avian groups. For example, members of the Ricinidae are known to infect hummingbirds and small Passeriformes, whereas members of the Menoponidae are widely distributed across most avian families Rózsa and Vas (2015). Similarly, we found lice from the genus *Ricinus* on 36 species of Passeriformes from 11 host families. *Myrsidea* is a broadly distributed, mega-diverse genus Valim and Weckstein (2013), found mostly on Passeriformes and is considered to have a high degree of host-specificity Price and Dalgleish (2007). We also found that the louse genus *Myrsidea*, from the family Menoponidae, was distributed on 120 bird species, two of which were non-Passeriformes.

In Ischnocera, the family Philopteridae is widely distributed on birds Rózsa and Vas (2015). The various genera of Philopteridae are often specialised morphologically and behaviourally for living on a single microhabitat in the plumage (e.g. wing, head and/or body feathers) where lice can avoid host preening Johnson et al. (2012). This microhabitat specialisation may in part explain the host specificity and diversity of these lice. The three most common genera of Philopteridae found in our study were *Philopterus*, *Brueelia* and *Rallicola.* Of these, *Philopterus* was the most widely distributed genus in this family, occurring on a diverse array of passerine host families and a single non-passerine host species (42 bird species). *Brueelia*, the most speciose genus of lice in the family Philopteridae, infects avian hosts from many orders, including Coraciiformes, Passeriformes and Piciformes Valim and Weckstein (2011) Valim and Weckstein (2013) Gustafsson and Bush (2017). Similarly, we found *Brueelia* on 30 bird species, including two species of Coraciiformes, two species of Piciformes and 26 species of Passeriformes. Finally, the third most frequently collected genus was *Rallicola*, found on 18 bird species, including one host species in the order Charadriiformes and 17 host species in the order Passeriformes. *Rallicola* is one of the most speciose of ischnoceran louse genera and has been reported from the avian host orders Apterygiformes, Charadriiformes, Gruiformes and Passeriformes Price et al. (2003), Smith (2001).

Thirty percent of the Colombian birds sampled (138 host species) were infected with lice. In Peru, Clayton et al. (1992) found that 48% of birds examined (127 host species) were infected by lice, whereas in Brazil, Marini et al. (1996) and Oniki (1999) found that 20% of 313 individual birds (53 species) and 63% of 60 birds (38 species) had lice, respectively. Enout et al. (2012) found that 65% of 57 avian host species sampled were infected with lice. These studies suggest that louse prevalence may vary geographically. For example, for the flycatcher, *Leptopogon amaurocephalus*, in Brazil, Marini et al. (1996) and Oniki (1999), sampled two and one individual hosts respectively and all were infected with lice, whereas Enout et al. (2012) found two of three individuals sampled infected with lice. We found that in Remedios, Colombia, only 16.6% (n=12) of *L. amaurocephalus* individuals were infected. However, other host species had similar prevalence rates as reported in previous studies. For example, in Brazil, Oniki (1999) found that 67% of *Turdus leucomelas* sampled (n=3) were infected with lice and we found that all individuals of *Turdus leucomelas* sampled at two localities by us (n=4) were infected. However, a second Brazilian study conducted by Enout et al. (2012) found a 43% infestation rate (n=35) for the same bird species. It is difficult to determine the drivers behind variation in prevalence. It is possible that we are seeing an ecological pattern due to differences in humidity at the different sampling localities Moyer et al. (2002), Bush et al. (2009), host distributions or due to the different methods used by researchers to collect the lice. Additional work, examining sites where lice were collected with the same methodology, will help to address these issues.

## Conclusions

This manuscript presents data on avian lice from 210 host species. We report and document significant new host-louse association records from poorly sampled yet diverse regions of the world. This information provides an important basis for future studies in the tropics and further enriches our knowledge of the parasite fauna associated with Neotropical birds.

## Supplementary Material

Supplementary material 1 Lice from Colombian and Peruvian birds Data type: Taxonomic
File: oo_159704.xlsx Juliana Soto-Patiño, Gustavo A Londoño, Jorge Enrique Avendaño, Jill E Jankowski, Andrew T Cook and Julie Allen

## Figures and Tables

**Figure 1a.**
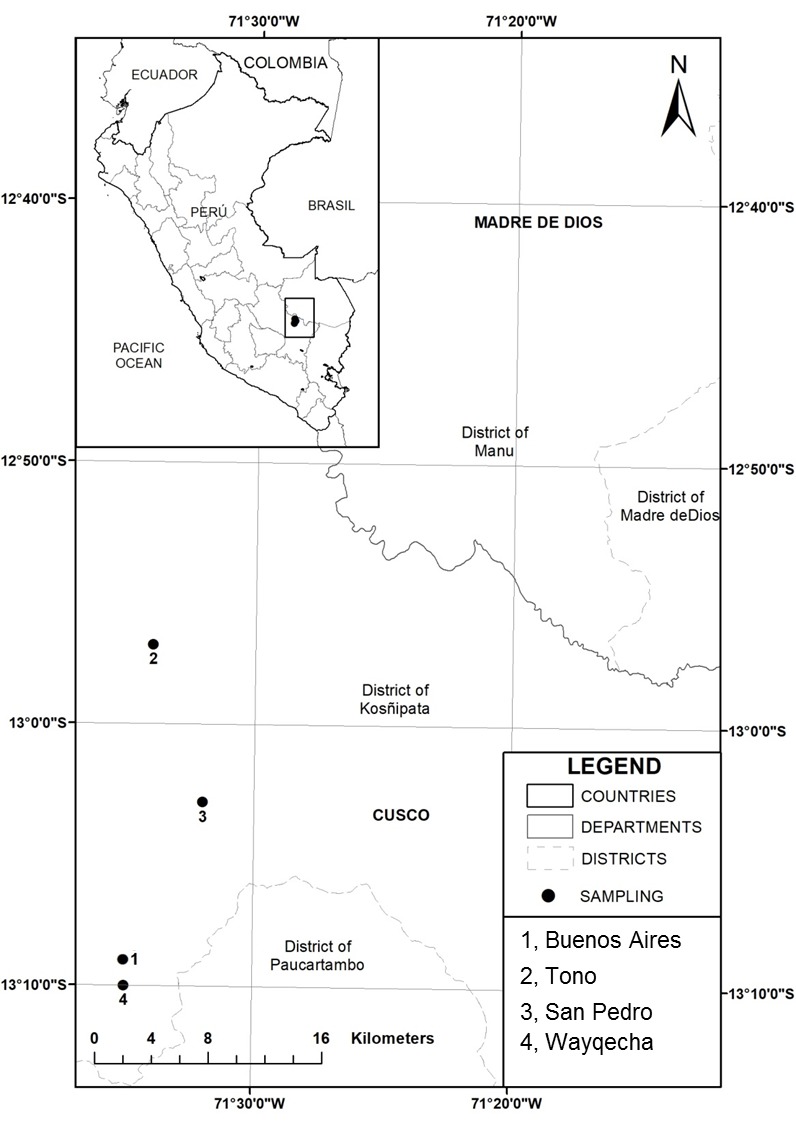
Peru

**Figure 1b.**
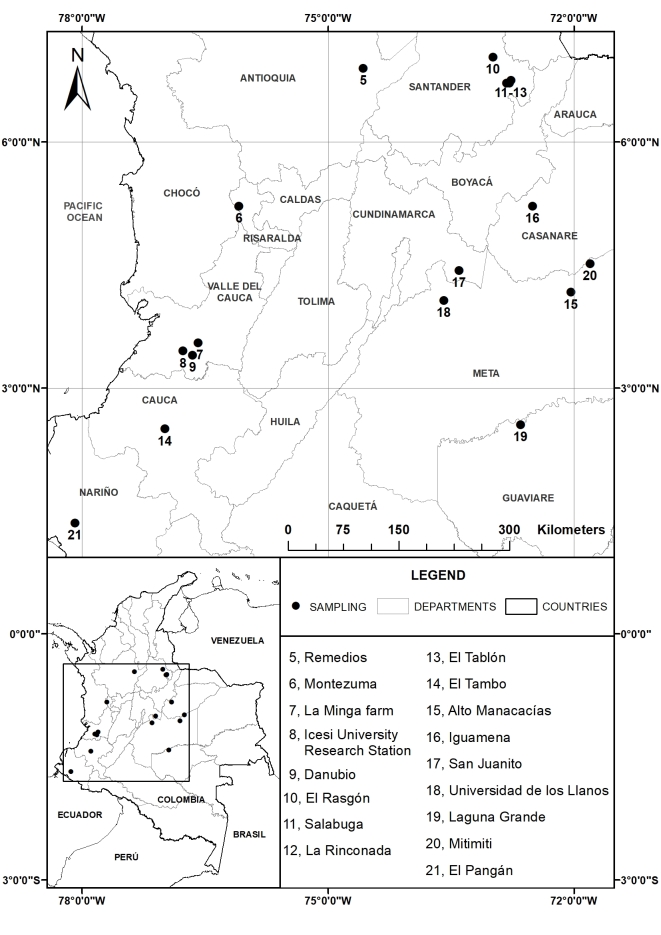
Colombia

**Table 1.** Sampling localities in Peru (2006–2007) and Colombia (2009–2016).

**Country**	**Department**	**Locality**	**Coordinates**	**Elevation (m a.s.l)**	**Habitat**	**Collector (s)**
Peru	Cusco	**1.** Buenos Aires (Mun. Paucartambo)	13°9'S, 71°35'W	2480-2550	Highland cloud forest	GAL-JEJ
	Cusco	**2.** Tono (Mun. Patria)	12°57'S, 71°34'W	800-1100	Andean foothill forest	GAL-JEJ
	Cusco	**3.** Lodge Gallito de las rocas (Mun. San Pedro)	13°03'S, 71°32'W	1200-1500	Montane cloud forest	GAL-JEJ
	Cusco	**4.** Wayqecha Biological Station (Mun. Paucartambo)	13°10'S, 71°35'W	2600-3000	Highland cloud forest	GAL-JEJ
Colombia	Santander	**5.** El Rasgón Reserve (Mun. Piedecuesta)	07°02'N, 72°59'W	2200	Primary cloud forest and borders	JEA
	Antioquia	**6.** Remedios (Mun. Remedios)	06°54'N, 74°34'W	500	Lowland humid forest	GAL
	Santander	**7.** Salabuga farm (Mun. San Andrés)	06°45'N, 72°46'W	2650	Primary cloud forest and borders	JEA
	Santander	**8.** El Tablón farm (Mun. San Andrés)	06°43'N, 72°49'W	2770-2800	Primary cloud forest and borders	JEA
	Santander	**9.** La Rinconada farm (Mun. San Andrés)	06°43'N, 72°47'W	2880	Primary cloud forest and borders	JEA
	Risaralda	**10.** Montezuma, Tatama Nationla Park (Mun. Pueblo Rico)	05°13'N, 76°05'W	1200-2500	Forest types from foothills, to mid and high elevation cloud forests	GAL
	Casanare	**11.** El Porvernir farm (Mun. Aguazul)	05°13'N, 72°30'W	350-400	Secondary humid tropical forest	JEA
	Meta	**12.** Universidad de los Llanos (Mun. Villavicencio)	04°4'N, 73°35'W	400-440	Secondary humid tropical forest	JEA
	Meta	**13.** Mitimiti farm (Mun. Puerto Gaitán)	04°31'N, 71°48'W	141	Savannah, gallery forest	JEA
	Cundinamarca	**14.** San Antonio farm (Mun. Medina)	04°26'N, 73°24'W	570	Secondary humid tropical forest	JEA
	Meta	**15.** Manacacías farm (Mun. Puerto Gaitán)	04°10'N, 72°02'W	200-250	Savannah, gallery forest	JEA
	Valle del Cauca	**16.** La Minga farm (Mun. La Cumbre)	03°33'N, 76°35'W	2000	Cloud forest on top of the Western cordillera	GAL
	Valle del Cauca	**17.** Icesi University research station, Zygia, Farallones de Cali National Park (Mun. Cali)	03°27'N, 76°46'W	2400	High elevation cloud forest	GAL
	Valle del Cauca	**18.** Danubio (Mun. Cali)	03°24'N, 76°39'W	2200	High elevation cloud forest	GAL-JSP
	Guaviare	**19.** Laguna Grande (Mun. San José del Guaviare)	02°33'N, 72°39'W	400	Savannah, gallery forest	JEA
	Cauca	**20.** Mirabilis-Swarovski Reserve (Mun. El Tambo)	02°31'N, 76°59'W	2270	Primary humid montante forest	JEA
	Cauca	**21.** Tambito Reserve (Mun. El Tambo)	02°30'N, 76°59'W	1500	Primary premontane forest	JEA
	Nariño	**22.** El Pangán Reserve (Mun. Barbacoas)	01°21'N, 78°05'W	710	Primary humid tropical forest	JEA

**Table 2.** Louse-host associations from birds captured in Colombia. N - number of birds examined, *Ni* - Number of infected birds. Superscripts A and I represent louse suborders Amblycera or Ischnocera and * indicates a previously unrecorded louse host association.

**Bird taxa**	**Louse genera**	**N**	***Ni***
** Tinamiformes **			
Tinamidae (1)			
*Crypturellus soui*	*Strongylocotes* sp. ^*I*^	1	1
** Galliformes **			
Odontophoridae (1)			
*Colinus cristatus*	*Gonioides* sp.*^I^*	2	2
	*Lipeurus* sp.*^I^*		
	*Oxylipeurus* sp.*^I^*		
** Columbiformes **			
Columbidae (3)			
*Leptotila rufaxilla*	*Columbicola* sp.*^I^*	2	2
	*Physconelloides* sp.*^I^*		
*Columbina talpacoti*	*Columbicola* sp.*^I^*	2	2
	*Physconelloides* sp.*^I^*		
*Zentrygon frenata*	*Campanulotes* sp.*^I^*	1	1
** Cuculiformes **			
Cuculidae (2)			
*Crotophaga ani*	*Osborniella* sp.*^A^*	1	1
	*Vernoniella* sp.*^I^*		
*Piaya cayana*	*Cuculoecus* sp.*^I^*	1	1
** Caprimulgiformes **			
Caprimulgidae (2)			
*Systellura longirostris*	*Mulcticola* sp.^I,^*	1	1
*Nyctidromus albicollis*	*Mulcticola* sp.*^I^*	3	3
** Apodiformes **			
Apodidae (1)			
*Chaetura meridionalis*	*Eureum* sp.^A,^*	1	1
Trochilidae (2)			
*Anthracothorax nigricollis*	*Trochiliphagus* sp.*^A^*	2	2
*Thalurania colombica*	*Myrsidea* sp. ^A,^*	1	1
** Charadriiformes **			
Scolopacidae (1)			
*Gallinago paraguaiae*	*Saemundssonia* sp. *^I^*	1	1
	*Rhynonirmus* sp.^I,^*		
Jacanidae (1)			
*Jacana jacana*	*Rallicola* sp.*^I^*	1	1
** Accipitriformes **			
Accipitridae (1)			
*Accipiter striatus*	*Degeeriella* sp.*^I^*	1	1
** Coraciiformes **			
Alcedinidae (3)			
*Chloroceryle americana*	*Alcedoffula* sp. *^I^*	2	2
*Chloroceryle inda*	*Alcedoffula* sp. *^I^*	2	2
*Chloroceryle aenea*	*Alcedoffula* sp. *^I^*	3	2
Momotidae (2)			
*Momotus momota*	*Philopterus* sp. *^I^*	2	2
*Momotus aequatorialis*	Brueelia s.l. ^I,^*	4	1
Galbuliformes			
Bucconidae (1)			
*Hypnelus ruficollis*	*Picicola* sp. ^I,^*	3	3
** Piciformes **			
Capitonidae (1)			
*Eubucco bourcierii*	*Penenirmus* sp.*^I^*	3	1
*Ramphastidae (1)*			
*Aulacorhynchus haematopygus*	*Austrophilopterus* sp.*^I^*	4	1
Picidae (6)			
*Picumnus squamulatus*	*Penenirmus* sp.^I,^*	1	1
*Melanerpes formicivorus*	*Penenirmus* sp.*^I^*	1	1
*Melanerpes rubricapillus*	Brueelia s.l. ^I,^*	1	1
*Picoides fumigatus*	Brueelia s.l. ^I,^*	5	1
*Colaptes rubiginosus*	*Penenirmus* sp. ^I,^*	1	1
*Dryocopus lineatus*	*Picicola* sp.^I,^*	1	1
** Psittaciformes **			
Psittacidae (3)			
*Brotogeris cyanoptera*	*Psittacobrosus* sp.*^A^*	1	1
*Forpus conspicillatus*	*Psittacobrosus* sp. *^A^*	1	1
*Eupsittula pertinax*	*Psittacobrosus* sp. *^A^*	2	2
	*Paragoniocotes* sp.*^I^*		
** Passeriformes **			
Thamnophilidae (3)			
*Dysithamnus puncticeps*	*Ricinus* sp.^A,^*	1	1
*Myrmotherula schisticolor*	*Ricinus* sp.^A,^*	6	1
*Formicivora grisea*	*Myrsidea* sp.^A,^*	1	1
Conopophagidae (1)			
*Conopophaga castaneiceps*	*Formicaphagus* sp.^I,^*	1	1
Grallaridae (1)			
*Grallaria alleni*	*Picicola* sp.^I,^*	1	1
Rhynocrhyptidae (1)			
*Scytalopus griseicollis*	*Rallicola* sp.^I,^*	3	1
Furnariidae (12)			
*Dendrocincla fuliginosa*	*Rallicola* sp.*^I^*	10	2
	*Ricinus* sp.^A,^*		
*Glyphorhynchus spirurus*	*Rallicola* sp.*^I^*	23	1
*Xiphorhynchus obsoletus*	*Rallicola* sp.^I,^*	2	2
*Dendroplex picus*	*Rallicola* sp.^I,^*	3	2
*Anabacerthia variegaticeps*	*Philopterus* sp.^I,^*	1	1
*Syndactyla subalaris*	*Rallicola* sp. ^I,^*	8	1
	*Myrsidea* sp.*^A^*		
*Clibanornis rubiginosus*	*Rallicola* sp.^I,^*	2	2
*Thripadectes ignobilis*	*Rallicola* sp.^I,^*	2	2
*Thripadectes virgaticeps*	*Rallicola* sp.^I,^*	4	2
	*Myrsidea* sp.*^A^*		
*Premnoplex brunnescens*	*Rallicola* sp.*^I^*	10	1
	*Myrsidea* sp.*^A^*		
*Cranioleuca vulpina*	*Rallicola* sp.^I,^*	1	1
	*Myrsidea* sp.*^A^*		
*Synallaxis unirufa*	*Rallicola* sp.^I,^*	2	1
Tyrannidae (13)			
*Elaenia flavogaster*	*Myrsidea* sp.*^A^*	1	1
*Elaenia parvirostris*	*Ricinus* sp.^A,^*	1	1
*Elaenia chiriquensis*	*Menacanthus* sp.^A,^*	1	1
*Elaenia pallatangae*	*Myrsidea* sp.^A,^*	2	1
*Mecocerculus leucophrys*	*Menacanthus* sp.^A,^*	2	1
*Mionectes striaticollis*	*Myrsidea* sp.*^A,^*	28	12
	*Philopterus* sp. ^I,^*		
*Mionectes olivaceus*	*Myrsidea* sp.*^A^*	13	4
	*Philopterus* sp.^I,^*		
*Mionectes oleagineus*	*Myrsidea* sp.*^A^*	18	2
*Leptopogon amaurocephalus*	*Philopterus* sp.^I,^*	16	3
*Atalotriccus pilaris*	*Philopterus* sp.^I,^*	1	1
*Rhynchocyclus olivaceus*	*Myrsidea* sp.*^A^*	4	1
*Platyrinchus coronatus*	*Myrsidea* sp.^A,^*	2	1
*Myiarchus tyrannulus*	*Philopterus* sp.^I,^*	1	1
Cotingidae (3)			
*Pipreola riefferii*	*Cotingacola* sp.*^I^*	26	8
	*Philopterus* sp. ^I,^*		
	*Myrsidea* sp.^A,^*		
*Pipreola arcuata*	*Pseudocophorus* sp.*^I^*	1	1
*Pipreola jucunda*	*Ricinus* sp.^A,^*	1	1
Pipridae (4)			
*Chloropipo flavicapilla*	*Philopterus* sp.^I,^*	7	2
*Manacus manacus*	*Ricinus* sp.*^A^*	9	2
	*Philopterus* sp.*^I^*		
*Pipra filicauda*	*Ricinus* sp.^A,^*	7	6
	*Philopterus* sp.^I,^*		
	*Myrsidea* sp.^A,^*		
*Machaeropterus regulus*	*Ricinus* sp.*^A^*	21	3
Tytiridae (1)			
*Pachyramphus polychopterus*	*Myrsidea* sp.^A,^*	3	3
	*Ricinus* sp.*^A^*		
Corvidae (1)			
*Cyanocorax violaceus*	*Brueelia s.l.^I^*	1	1
	*Myrsidea* sp.*^A^*		
Hirundinidae (1)			
*Progne tapera*	*Philopterus* sp.^I,^*	2	2
	*Myrsidea* sp.*^A^*		
Troglodytidae (2)			
*Troglodytes aedon*	*Penenirmus* sp.*^I^*	6	2
*Cyphorhinus thoracicus*	*Penenirmus* sp.^I,^*	8	1
Turdidae (10)			
*Myadestes ralloides*	*Philopterus* sp.^I,^*	29	15
	*Myrsidea* sp.^A,^*		
*Catharus ustulatus*	*Philopterus* sp.^I,^*	10	4
	*Myrsidea* sp.*^A^*		
*Entomodestes coracinus*	Brueelia s.l.^I,^*	8	4
	*Myrsidea* sp.*^A^*		
	*Myrsidea* sp.*^A^*		
*Turdus leucops*	Brueelia s.l.^I,^*	13	2
*Turdus leucomelas*	*Myrsidea* sp.*^A^*	4	4
	*Brueelia s.l.^I^*		
*Turdus nudigenis*	*Myrsidea* sp.*^A^*	6	6
	*Brueelia s.l.^I,+^*		
*Turdus ignobilis*	*Myrsidea* sp.*^A^*	1	1
	Brueelia s.l.^I,^*		
*Turdus fuscater*	*Myrsidea* sp.^A,^*	2	1
	Brueelia s.l.^I,^*		
*Turdus serranus*	*Myrsidea* sp.^A,^*	19	12
	Brueelia s.l.^I,^*		
*Turdus albicollis*	*Myrsidea* sp.^A,^*	2	2
	Brueelia s.l. ^I,^*		
Thraupidae (34)			
*Paroaria nigrogenis*	*Myrsidea* sp.^A,^*	1	1
	Brueelia s.l.^I,^*		
*Schistochlamys melanopis*	*Myrsidea* sp.*^A^*	1	1
*Hemispingus atropileus*	*Myrsidea* sp.^A,^*	2	1
*Hemispingus frontalis*	*Myrsidea* sp.^A,^*	7	1
*Ramphocelus carbo*	*Myrsidea* sp.*^A^*	15	15
	Brueelia s.l.^I,^*		
	*Ricinus* sp.*^A^*		
*Ramphocelus flammigerus*	*Myrsidea* sp.^A,^*	3	2
*Bangsia edwardsi*	*Myrsidea* sp.^A,^*	2	2
*Bangsia aureocincta*	*Philopterus* sp.^I,^*	3	1
	*Myrsidea* sp.^A,^*		
*Buthraupis montana*	*Myrsidea* sp.^A,^*	2	2
*Chlorornis riefferii*	*Myrsidea* sp.^A,^*	3	1
*Anisognathus somptuosus*	*Myrsidea* sp.^A,^*	10	6
*Iridosornis rufivertex*	*Myrsidea* sp.^A,^*	1	1
*Chlorochrysa phoenicotis*	*Myrsidea* sp.^A,^*	4	2
*Thraupis palmarum*	*Myrsidea* sp.*^A^*	3	2
	*Ricinus* sp.*^A^*		
*Thraupis cyanocephala*	*Myrsidea* sp.*^A^*	3	2
	Brueelia s.l.^I,^*		
*Tangara heinei*	*Myrsidea* sp.^A,^*	4	2
*Tangara cayana*	*Myrsidea* sp.*^A^*	9	9
	*Machaerilaemus* sp.^A,^*		
*Tangara vitriolina*	*Myrsidea* sp.^A,^*	1	1
*Tangara rufigula*	*Myrsidea* sp.^A,^*	3	3
*Tangara nigroviridis*	*Myrsidea* sp.^A,^*	5	1
*Tangara gyrola*	*Myrsidea* sp.*^A^*	1	1
*Tangara arthus*	*Myrsidea* sp.^A,^*	8	1
*Tangara icterocephala*	*Myrsidea* sp.*^A^*	3	3
	*Ricinus* sp.^A,^*		
*Tersina viridis*	*Menacanthus* sp.^A,^*	1	1
*Diglossa albilatera*	*Myrsidea* sp.^A,^*	14	2
*Diglossa caerulescens*	*Myrsidea* sp.^A,^*	4	2
	*Philopterus* sp.*^I^*		
*Catamblyrhynchus diadema*	*Myrsidea* sp.^A,^*	3	2
*Haplospiza rustica*	*Philopterus* sp.^I,^*	2	1
*Saltator maximus*	*Myrsidea* sp.*^A^*	2	2
*Saltator coerulescens*	*Myrsidea* sp.^A,^*	1	1
*Volatinia jacarina*	*Myrsidea* sp.^A,^*	2	2
*Sporophila minuta*	*Ricinus* sp.^A,^*	1	1
*Sporophila crassirostris*	*Philopterus* sp.^I,^*	1	1
*Coereba flaveola*	Brueelia s.l.^I,^*	1	1
Emberizidae (6)			
*Oreothraupis arremonops*	*Myrsidea* sp.^A,^*	3	1
*Chlorospingus flavigularis*	*Myrsidea* sp.^A,^*	3	2
*Chlorospingus flavopectus*	*Myrsidea* sp.^A^	10	9
	*Ricinus* sp. ^A,^*		
	*Philopterus* sp. ^I,^*		
	*Penenirmus* sp.^I,^*		
*Chlorospingus semifuscus*	*Myrsidea* sp.^A,^*	8	5
	*Philopterus* sp.^I,^*		
	*Brueelias.l.*^I,^*		
*Arremonops conirostris*	*Myrsidea* sp.^A^	3	3
*Arremon brunneinucha*	*Myrsidea* sp.^A^	18	8
	* Brueelia * *s.l.* ^I^		
Cardinalidae (1)			
*Habia cristata*	*Myrsidea* sp.^A,^*	1	1
	*Brueelias.l.*^I,^*		
Parulidae (5)			
*Setophaga fusca*	*Ricinus* sp.^A^	2	1
*Myiothlypis fulvicauda*	*Menacanthus* sp.^A,^*	1	1
*Myiothlypis coronata*	*Myrsidea* sp.^A,^*	17	7
	*Brueelias.l.*^I,^*		
*Basileuterus tristriatus*	*Myrsidea* sp.^A,^*	18	4
	*Menacanthus* sp. ^A,^*		
	*Myrsidea* sp.^A^		
Myioborus miniatus	*Ricinus* sp.^A,^*	7	2
*Icteridae* (4)			
*Psarocolius decumanus*	*Myrsidea* sp.^A^	1	1
*Cacicus cela*	*Myrsidea* sp.^A^	1	1
	* Brueelia * *s.l.* ^I^		
*Cacicus chrysonotus*	*Myrsidea* sp.^A,^*	4	1
	* Brueelia * *s.l.* ^I^		
*Gymnomystax mexicanus*	*Myrsidea* sp.^A,^*	1	1
Fringillidae (3)			
*Euphonia chlorotica*	*Myrsidea* sp.^A,^*	1	1
*Euphonia laniirostris*	*Myrsidea* sp.^A^	1	1
*Chlorophonia pyrrhophrys*	*Philopterus* sp.^I,^*	1	1
	*Brueelias.l.*^I,^*		
TOTAL (138)		641	310

**Table 3.** Host-louse associations from sites in Peru. *Ni* Number of birds infested. Superscripts *A* and *I* represent the suborders of lice Amblycera and Ischnocera, * represents new host-louse association reported in this study. ***^+^***New genus reported for a host species with louse associations known *(No)* Number of host species representing each bird family.

**Bird taxa**	**Louse genera**	***Ni***
** Columbiformes **		
Columbidae (1)		
*Geotrygon montana*	*Columbicola* sp.*^I^*	1
** Apodiformes **		
Trochilidae (2)		
*Coeligena violifer*	*Trochiloecetes* sp.*^A,^* *	1
*Thalurania furcata*	*Trochiliphagus* sp.^A,^*	1
** Coraciiformes **		
Momotidae (1)		
*Baryphthengus martii*	*Brueelia s.l.^I^*	1
** Piciformes **		
Capitonidae (1)		
*Eubucco versicolor*	*Myrsidea* sp.*^A,^* *	1
** Passeriformes **		
Thamnophilidae (7)		
*Thamnophilus caerulescens*	*Formicaphagus* sp.*^I,^* *	1
	*Macharilaemus* sp.*^A,^* *	
*Dysithamnus mentalis*	*Formicaphagus* sp.*^I,^* *	3
	*Myrsidea* sp.*^A,^* *	
*Pyriglena leuconota*	*Formicaphagus* sp.*^I^*	1
*Myrmoborus myotherinus*	*Formicaphagus* sp.*^I,^* *	1
*Sciaphylax hemimelaena*	*Ricinus* sp.*^A,^* *	1
*Rhegmatorhina melanosticta*	*Ricinus* sp.*^A,^* *	1
	*Myrsidea* sp.*^A,^* *	
*Phlegopsis nigromaculata*	*Myrsidea* sp.*^A,^* *	1
Grallaridae (1)		
*Grallaricula flavirostris*	*Myrsidea* sp.*^A,^* *	1
Formicariidae (1)		
*Chamaeza campanisona*	*Myrsidea* sp.*^A,^* *	1
Furnariidae (15)		
*Dendrocincla fuliginosa*	*Rallicola* sp.*^I^*	1
*Glyphorhynchus spirurus*	*Myrsidea* sp.*^A^*	3
	*Rallicola* sp.*^I^*	
*Xiphocolaptes promeropirhynchus*	*Rallicola* sp.*^I,^* *	1
*Xiphorhynchus triangularis*	*Rallicola* sp.*^I^*	2
*Anabacerthia striaticollis*	*Philopterus* sp.^I,^*	3
	*Ricinus* sp.^A,^*	
*Syndactyla ucayalae*	*Myrsidea* sp.*^A,^* *	1
*Clibanornis rubiginosus*	*Myrsidea* sp.*^A,^* *	2
	*Rallicola* sp.*^I^*	
*Thripadectes holostictus*	*Furnariphilus* sp.*^I,^* *	2
	*Myrsidea* sp.*^A,^* *	
*Thripadectes melanorhynchus*	*Myrsidea* sp.*^A,^* *	5
	*Rallicola* sp.*^I^*	
*Automolus ochrolaemus*	*Myrsidea* sp.*^A^*	2
*Automolus subulatus*	*Myrsidea* sp.*^A,^* *	1
	*Rallicola* sp.*^I^*	
*Premnoplex brunnescens*	*Myrsidea* sp.*^A^*	1
*Margarornis squamiger*	*Rallicola* sp.*^I^*	1
*Asthenes helleri*	*Philopterus* sp.*^I,^* *	2
*Synallaxis azarae*	*Furnariphilus* sp.*^I,^* *	1
Tyrannidae (16)		
*Phylloscartes poecilotis*	*Myrsidea* sp.*^A,^* *	1
*Phylloscartes ophtalmicus*	*Philopterus* sp.*^I,^* *	1
	*Myrsidea* sp.*^A^*	
*Mionectes olivaceus*	*Myrsidea* sp.*^A^*	17
*Mionectes striaticollis*	*Myrsidea* sp.*^A^*	26
	*Philopterus* sp.*^I,^* *	
*Mionectes oleagineus*	*Myrsidea* sp.*^A^*	6
*Leptopogon superciliaris*	*Myrsidea* sp.*^A,^* *	7
	*Philopterus* sp.*^I^*	
*Myiotriccus ornatus*	*Myrsidea* sp.*^A,^* *	1
*Lophotriccus pileatus*	*Philopterus* sp.*^I,^* *	1
*Myiophobus inornatus*	*Ricinus* sp.*^A,^* *	1
*Pyrrhomyias cinnamomeus*	*Philopterus* sp.*^I,^* *	1
*Mitrephanes olivaceus*	*Philopterus* sp.*^I,^* *	1
*Ochthoeca frontalis*	*Philopterus* sp.*^I,^* *	6
	*Myrsidea* sp.*^A^*	
*Ochthoeca pulchella*	*Philopterus* sp.*^I,^* *	6
	*Myrsidea* sp.*^A,^* *	
*Ochthoeca cinnamomeiventris*	*Philopterus* sp.*^I,^* *	1
*Ochthoeca rufipectoralis*	*Philopterus* sp.*^I,^* *	2
*Conopias cinchoneti*	*Philopterus* sp.*^I,^* *	1
Cotingidae (2)		
*Pipreola intermedia*	*Myrsidea* sp.*^A,^* *	2
	*Philopterus* sp.*^I,^* *	
*Pipreola pulchra*	*Myrsidea* sp.*^A,^* *	1
Pipridae (4)		
*Chiroxiphia boliviana*	*Myrsidea* sp.*^A,^* *	6
	*Philopterus* sp.*^I^*	
	*Ricinus* sp.*^A^*	
*Lepidothrix coeruleocapilla*	*Myrsidea* sp.*^A,^* *	8
	*Philopterus* sp.*^I,^* *	
	*Ricinus* sp.*^A,^* *	
*Pipra fasciicauda*	*Myrsidea* sp.*^A,^* *	1
	*Philopterus* sp.*^I,^* *	
*Machaeropterus pyrocephalus*	*Philopterus* sp.*^I,^* *	2
	*Ricinus* sp.*^A^*	
Troglodytidae (1)		
*Henicorhina leucophrys*	*Myrsidea* sp.*^A,^* *	1
Turdidae (6)		
*Myadestes ralloides*	*Myrsidea* sp.*^A,^* *	4
	*Philopterus* sp.*^I,^* *	
	*Brueelia s.l.^I,^* *	
*Catharus ustulatus*	*Myrsidea* sp.*^A^*	5
	*Brueelia s.l.^I^*	
*Entomodestes leucotis*	*Myrsidea* sp.*^A,^* *	4
	*Brueelia s.l.^I^*	
	*Sturnidoecus* sp.*^I,^* *	
*Turdus leucops*	*Myrsidea* sp.*^A,^* *	1
*Turdus fuscater*	*Myrsidea* sp.*^A,^* *	1
	*Philopterus* sp.*^I^*	
*Turdus serranus*	*Myrsidea* sp.*^A,^* *	3
	*Menacanthus* sp.*^A^*	
	*Philopterus* sp.*^I^*	
	*Brueelia s.l.^I,^* *	
	*Ricinus* sp.*^A^*	
Thraupidae (25)		
*Hemispingus superciliaris*	*Ricinus* sp.*^A,^* *	1
*Hemispingus melanotis*	*Myrsidea* sp.*^A,^* *	4
	*Ricinus* sp.*^A^*	
*Trichothraupis melanops*	*Myrsidea* sp.*^A,^* *	3
*Thlypopsis ruficeps*	*Philopterus* sp.*^I,^* *	2
	*Ricinus* sp.*^A^*	
*Ramphocelus carbo*	*Myrsidea* sp.*^A^*	2
*Buthraupis montana*	*Myrsidea* sp.*^A,^* *	1
*Chlorornis riefferii*	*Myrsidea* sp.*^A,^* *	1
*Iridosornis analis*	*Myrsidea* sp.*^A,^* *	
	*Brueelia s.l.^I,^* *	5
*Iridosornis jelskii*	*Myrsidea* sp.*^A,^* *	
*Chlorochrysa calliparaea*	*Myrsidea* sp.*^A,^* *	1
*Thraupis cyanocephala*	*Myrsidea* sp.*^A^*	1
*Tangara cyanicollis*	*Myrsidea* sp.*^A,^* *	1
	*Brueelia s.l.^I,^* *	2
*Tangara punctata*	*Myrsidea* sp.*^A,^* *	
*Tangara nigroviridis*	*Myrsidea* sp.*^A,^* *	2
*Tangara chilensis*	*Myrsidea* sp.*^A^*	2
*Tangara gyrola*	*Myrsidea* sp.*^A^*	1
*Tangara schrankii*	*Myrsidea* sp.*^A,^* *	1
*Tangara arthus*	*Myrsidea* sp.*^A,^* *	2
*Conirostrum albifrons*	*Ricinus* sp.*^A,^* *	2
*Diglossa mystacalis*	*Myrsidea* sp.*^A,^* *	2
*Diglossa brunneiventris*	*Myrsidea* sp.*^A,^* *	2
*Diglossa glauca*	*Myrsidea* sp.*^A,^* *	1
	*Ricinus* sp.*^A,^* *	1
*Diglossa cyanea*	*Myrsidea* sp.*^A,^* *	
*Saltator maximus*	*Brueelia s.l.^I,+^*	3
*Coereba flaveola*	*Myrsidea* sp.*^A^*	1
Emberizidae (6)		
*Chlorospingus flavigularis*	*Myrsidea* sp.*^A,^* *	8
*Chlorospingus parvirostris*	*Myrsidea* sp.*^A,^* *	2
*Chlorospingus flavopectus*	*Myrsidea* sp.*^A^*	1
*Arremon taciturnus*	*Myrsidea* sp.*^A^*	2
*Arremon brunneinucha*	*Brueelia s.l.^I^*	1
*Atlapetes melanolaemus*	*Ricinus* sp.*^A,^* *	4
Cardinalidae (1)		
*Piranga leucoptera*	*Myrsidea* sp.*^A,^* *	1
Parulidae (5)		
*Myiothlypis luteoviridis*	*Myrsidea* sp.*^A,^* *	5
	*Ricinus* sp.*^A,^* *	
*Myiothlypis signata*	*Myrsidea* sp.*^A,^* *	3
	*Menacanthus* sp.*^A,^* *	
	*Picicola* sp.*^I,^* *	
	*Ricinus* sp.*^A,^* *	
*Myiothlypis bivittata*	*Myrsidea* sp.*^A,^* *	5
	*Ricinus* sp.*^A^*	
*Myiothlypis coronata*	*Myrsidea* sp.*^A,^* *	7
	*Brueelia s.l.^I,^* *	
*Myioborus miniatus*	*Myrsidea* sp.*^A^*	6
	*Menacanthus* sp.*^A,^* *	
	*Ricinus* sp.*^A,^* *	
Icteridae (1)		
*Amblycercus holosericeus*	*Philopterus* sp.*^I,^* *	1
Fringillidae (2)		
*Euphonia mesochrysa*	*Ricinus* sp.*^A,^* *	1
*Euphonia xanthogaster*	*Myrsidea* sp.*^A,^* *	7
**TOTAL (98)**		**262**
